# Next-Generation Sequencing (NGS) in COVID-19: A Tool for SARS-CoV-2 Diagnosis, Monitoring New Strains and Phylodynamic Modeling in Molecular Epidemiology

**DOI:** 10.3390/cimb43020061

**Published:** 2021-07-30

**Authors:** Goldin John, Nikhil Shri Sahajpal, Ashis K. Mondal, Sudha Ananth, Colin Williams, Alka Chaubey, Amyn M. Rojiani, Ravindra Kolhe

**Affiliations:** 1 Department of Pathology, Medical College of Georgia, Augusta University, BAE 2576, 1120 15th Street, Augusta, GA 30912, USA; jgoldin@augusta.edu (G.J.); nsahajpal@augusta.edu (N.S.S.); amondal@augusta.edu (A.K.M.); sananth@augusta.edu (S.A.); COLWILLIAMS@augusta.edu (C.W.); achaubey@bionanogenomics.com (A.C.); 2 Bionano Genomics Inc., San Diego, CA 92121, USA; 3 Department of Pathology, Penn State University College of Medicine, Hershey, PA 16802, USA; arojiani@pennstatehealth.psu.edu

**Keywords:** next-generation sequencing, COVID-19, challenges, diagnostic assay

## Abstract

This review discusses the current testing methodologies for COVID-19 diagnosis and explores next-generation sequencing (NGS) technology for the detection of SARS-CoV-2 and monitoring phylogenetic evolution in the current COVID-19 pandemic. The review addresses the development, fundamentals, assay quality control and bioinformatics processing of the NGS data. This article provides a comprehensive review of the obstacles and opportunities facing the application of NGS technologies for the diagnosis, surveillance, and study of SARS-CoV-2 and other infectious diseases. Further, we have contemplated the opportunities and challenges inherent in the adoption of NGS technology as a diagnostic test with real-world examples of its utility in the fight against COVID-19.

## 1. Introduction

The outbreak of COVID-19 was first identified in Wuhan, China, a city in the Hubei province, in December 2019. A cluster of unexplained pneumonia cases led to the designation of COVID-19 as novel pneumonia, and work began immediately to identify the pathogen responsible for the outbreak and to delineate its genomic sequence [1,2,3,4]. The pathogen was identified as severe acute respiratory syndrome coronavirus-2 (SARS-CoV-2) and the sequence was first reported to the local authorities on 5 January 2020 and was released on the open-access virology website virological.org on 11 January [5].

The virus has now spread to all corners of the globe, and innovators in academia, government, and private industry are moving at an unprecedented scale and pace to bring forward solutions to mitigate and resolve the crisis. Of the several measures taken to control the spread of disease, testing for SARS-CoV-2 in the population is a primary measure that has been implemented globally. Most importantly, testing provides people with evidence of infection, allowing them and those they have encountered to take necessary precautions, such as quarantining, to reduce community exposure. Additionally, standard, widespread testing yields data for researchers and public health officials to utilize for transmission modeling and public policy decision making regarding issues such as social distancing and mask use, among others.

Next-generation sequencing (NGS) is a technology used by countless laboratories across the world for investigating the genetic makeup of all forms of living beings, but its utilization in infectious disease diagnostics is relatively scarce at the present moment. Information gleaned from NGS, whereby the pathogen’s genome sequence is determined, yields a much greater trove of knowledge than the data produced by standard testing procedures, including information for the development of therapeutics and vaccines, the monitoring of changes in the virus as it circulates through the population, and deeper insights into patterns of transmission across time and geography.

This review aims to serve as a reference guide to learn about the background, utilization, and implementation of NGS technology in the setting of infectious disease, with the SARS-CoV-2 pandemic serving as a backdrop. First, we reviewed the advantages and disadvantages of current diagnostic methods for COVID-19, followed by an introduction to NGS technology and its bioinformatic processes. Next, we reviewed the studies deploying NGS early on in the SARS-CoV-2 pandemic in an effort to highlight its myriad of overlapping applications in diagnostics, phylogenetic monitoring, and scientific inquiry. We concluded with a review of challenges and opportunities faced by laboratories and scientists as they work to implement NGS technology in their organizations. The mechanisms used by different manufacturers of NGS technology to generate raw data varies extensively, and a comprehensive review of the step-by-step process of read generation used by each company is outside the scope of this article. The current challenges and usage of NGS technology in the SARS-CoV-2 pandemic mentioned here are up to date as of early 2021.

## 2. Current Testing Methods

The majority of SARS-CoV-2 diagnostic testing procedures in use presently fall into two categories: molecular-based assays, ideal for detecting acute infection, and serological- or immunological-based assays, useful for detecting whether a patient has been previously infected [6]. Both camps have valid utility in the management of a pandemic. The rapid identification of acute infections is of paramount importance to slow the spread of the disease, while immunological-based methods are useful in yielding data regarding herd immunity, accurate large-scale statistics (including the true death rate), the possibility of reinfection, and identification of plasma donors. As is generally the case in times of upheaval, innovative methods are rapidly being developed, including a subset of diagnostic tools utilizing isothermal amplification technology.

### 2.1. Molecular-Based Testing Methods

Reverse-transcription polymerase chain reaction (RT-PCR) is the most commonly used diagnostic test for SARS-CoV-2, and it can detect the virus in saliva [7], blood [8], rectal swabs, urine, stool [9], nasopharyngeal swabs, oropharyngeal swabs, nasopharyngeal washes, nasal aspirates, sputum, bronchoalveolar lavage (BAL) fluid, and tracheal aspirates [10]. Its widespread use is attributed to its simplicity and relatively high sensitivity. 

RT-PCR assays reverse transcribe viral RNA into cDNA, and then amplify regions of the cDNA to levels sufficient to detect the presence of the pathogen. The process relies on DNA primer–probe sets complementary to specific regions of the SARS-CoV-2 cDNA, and labs around the world have been racing to create these sets since the first SARS-CoV-2 genome was shared publicly. Tests have been developed that target many SARS-CoV-2 specific regions, including the ORF1ab, nucleocapsid (N), spike (S), RNA-dependent RNA polymerase (RdRp), and envelope (E) gene regions [11]. These assays can be performed as one-step process or separately as two-step processes. The one-step process is faster and easier to perform, while the two-step process tends to be more sensitive as a result of better target amplification. The United States Center for Disease Control (CDC) designed a one-step diagnostic assay that can quantify viral load in real time during the amplification reaction (RT-PCR) [10]. 

Vogels et al. performed an analysis of the sensitivity and efficiency of some of the most common RT-PCR primer–probe sets used for the detection of SARS-COV-2 worldwide, examining primer–probe sets from the China CDC, United States CDC, Charité Institute of Virology (Charité), and Hong Kong University (HKU) [12]. They showed that most common RT-PCR primer–probe sets are similarly reliable (except for issues surrounding the RdRp-SARSr set from Charité). They showed that these assays were all (except for the RdRp-SARSr set) able to partially detect SARS-CoV-2 RNA at 10^0^ (1) copies per uL and reliably differentiate positive and negative samples at 100 SARS-CoV-2 RNA copies/uL. These results are relatively similar to those released by the United States CDC showing that their qRT-PCR diagnostic assay had a limit of detection of 10^0^–10^0.5^ (1–3.16) SARS-CoV-2 RNA copies per uL [13].

A newer iteration of PCR testing is digital PCR (dPCR), whereby samples are diluted and thousands of PCR reactions are run in parallel in separate wells. This process works using Poisson statistical theory, assuming that for each micro-PCR amplification well there are either 0 or 1 copies of the nucleic acid of interest, allowing absolute quantification of nucleic acid present in a sample [14].

The dPCR process uses the same primer sets as RT-PCR, making direct comparisons between assays relatively easy to perform. Tao et al., in Wuhan, China, has reported on the use of digital droplet PCR (ddPCR), a type of dPCR technology where a sample is divided into tens of thousands of consistent nanodroplets, and compared by RT-PCR [15]. They assessed the limit of detection for SARS-CoV-2 for both assay types in the laboratory and performed a double-blinded comparison between the two assay types using identical primer sets on 63 throat swab samples from suspected COVID-19 outpatients in Wuhan, China. The limit of detection of their RT-PCR and ddPCR assays was assessed using serially diluted samples and primer–probe sets for both the ORF1ab and N genes. The data showed around 500 times higher sensitivity of the ddPCR assay versus RT-PCR: 2.1 and 1.8 copies/reaction for ORF1ab and N in ddPCR versus 1039 and 873.2 copies/reaction for RT-PCR with the same primer–probe sets, respectively. They further probed the assays’ abilities with a double-blinded study of 63 suspected SARS-CoV-2 outpatients. Specimens were each tested by both assay types, and patients were subsequently monitored and re-tested by conventional methods over the following two weeks to allow for data gathering on whether they had the virus or not. The ddPCR testing proved much more reliable with a sensitivity of 94% (versus 40% for RT-PCR), a specificity of 100% (100%), positive predictive value (PPV) of 100% (100%), and negative predictive value (NPV) of 100% (16%). These results demonstrate the potential of ddPCR in viral diagnostics, especially in situations where the low viral load can be an issue such as with asymptomatic carriers of SARS-CoV-2. However, this study represents only one small-scale investigation, and ddPCR technology is still expensive and mostly out of reach for widespread adoption during the current pandemic.

### 2.2. Serological and Immunological-Based Testing Methods

Serological testing mainly utilizes blood serum or plasma for detection of specific IgM or IgG antibodies. IgM antibodies are usually produced first after initial infection, while IgG antibodies generally predominate after a few weeks following an infection. Antibody tests are not ideal for acute diagnosis of viral infections because there is usually a delay between infection and antibody generation, and some patients may not generate antibodies at all. Currently, there is no clear-cut evidence that the presence of antibodies provides immune protection from SARS-CoV-2 infection [6]. These issues warrant investigation in large-scale studies, and more generally, studies are needed to enumerate the human immune response to SARS-CoV-2 infection. Further research is needed to determine the duration of these antibodies and their viability in fighting off re-infections as well as the response of T-cells to infection. 

More specifically, most immunological-based assays probe for the presence of IgM or IgG antibodies against viral antigens such as subunits of the spike glycoprotein (S) or nucleocapsid protein (N) [6]. Other immunological assays deploy antibodies themselves to search for the presence of viral antigens in a patient’s sample. Some common serological assays include Enzyme-linked immunosorbent assays (ELISA), neutralization tests, and immunofluorescent assays [6,16]. Several antigen-based assays were employed in the first phase of the pandemic for rapid detection of SARS-CoV-2. Although the sensitivity of the antigen test is significantly lower compared to qPCR assays, they play a significant role as point-of-care tests and for rapid detection worldwide. 

ELISA testing is routinely used for the detection and/or quantification of various substances in the blood. When investigating whether antibodies are present in a sample, viral antigens are immobilized to “catch” patient antibodies, and reporter antibodies are used in detection and quantification. Similarly, immunofluorescent assays analyze fluorescent output when patient antibodies interact with viral proteins. In contrast to antigen–antibody capture assays, neutralization tests analyze the ability of patient blood samples to prevent viral replication in cultured cells to evaluate the presence and/or levels of antibodies therein. These tests are not suitable for mass SARS-CoV-2 diagnostics due to biosafety concerns, but they will prove to be vital for vaccine development [6,16].

Cai et al. have developed a peptide-based immunofluorescence assay that showed promising initial results. Samples from 276 RT-PCR confirmed COVID-19 patients demonstrated the presence of IgG and IgM against SARS-CoV-2 in 71.4% and 57.2% of patients, respectively. Overall, 225/276 (81.52%) patients tested positive [17], indicating that the assay could be a useful diagnostic procedure when used in conjunction with standard RT-PCR assays. However, there are several limitations to these findings, including the fact that all of the COVID-19 samples came from the hospital setting. These patients were likely experiencing more severe symptoms than the average infected population, and as such, their immune response was not likely to be indicative of the general population. 

With the rapidly evolving pandemic, many of these immunological assays have shown significant promise for utility in the SARS-CoV-2 detection pandemic and are key assays that have been implemented to address several imperative questions as to the duration for which the antibodies remain in the circulation upon natural infection compared to after vaccination. The data from several longitudinal studies that are monitoring the antibody levels and neutralization capacity after infection or vaccine are critical for our understanding and for framing recommendations to contain the spread of infection. 

### 2.3. New Developments

Several new diagnostic assays are being developed based on isothermal nucleic acid amplification, which, unlike RT-PCR, can operate at a constant temperature. This process eliminates the need for the expensive thermal cycling equipment necessary for PCR, a major bottleneck in testing in many countries, including the US. Reverse transcription loop-mediated isothermal amplification (RT-LAMP), transcription-mediated amplification (TMA), and CRISPR-based assays are a few of the techniques under development that utilize isothermal amplification [6] and could lead to new, rapid point-of-care testing. 

RT-LAMP technology presents an opportunity for fast and economical testing. Yang et al. have reported on an RT-LAMP assay for the detection of SARS-COV-2 [18]. Under their protocol, amplification is carried out in a single tube at 63 °C, and results are obtained within an hour. They developed and used primers for the *ORF1ab*, *E*, and *N* genes of the SARS-CoV-2 genome and analyzed nasopharyngeal samples from the Jinan CDC infectious disease hospital, reporting an accuracy of 99% for their assay. The sample size was limited (17 positive and 191 negative SARS-CoV-2 samples), but continued development of this technology could have substantial implications for developing nations due to the low cost and simplicity of this assay. Abbot Diagnostics currently offers the ID NOW COVID-19 test, which is based on RT-LAMP technology, as a rapid point-of-care test, and this test is being used in almost all states in the US [19]. These tests yield results within 13 min but are limited to a single sample per run, making high volume throughput difficult [6].

Transcription-mediated amplification is another iteration of isothermal amplification technology. The Panther Fusion SARS-CoV-2 test from Hologic has been issued Emergency Use Authorization (EUA) from the FDA and allows for high throughput testing (1000 tests per 24 h) as well as screening for other common respiratory viruses [20]. 

CRISPR-based assays are under development by Mammoth Biosciences and Sherlock Biosciences [6]. The potential benefits of these assays include fast turnaround time, ease of use, and lack of need for thermal cycling equipment. The Mammoth Biosciences DETECTR assay uses CRISPR-associated (Cas) enzymes to cleave a reporter RNA sequence followed by subsequent RT-LAMP isothermal amplification to detect the presence of SARS-CoV-2 RNA in respiratory swabs. A validation study performed on 36 confirmed COVID-19 patients and 42 patients with other respiratory illnesses in the US reported a 95% PPV and 100% NPV. Limitations of the study include a small sample size and an increased limit of detection (10 viral RNA copies per uL versus 1 viral copy per uL for the US CDC qRT-PCR test) [21].

While these novel approaches to diagnostics for SARS-CoV-2 infection are exciting and worthy of pursuit by governments and corporations, the vast majority of testing worldwide is still carried out with PCR-based systems. To date, 112 molecular-based assays for detection of SARS-CoV-2 are available in the US, and of them, 90% use PCR-based assays, 6% use isothermal amplification methods, 2% use hybridization techniques, and 2% use CRISPR technology [6].

PCR-based assays, while widespread and in common usage, have their own set of challenges. Problems with sampling techniques, nucleic acid extraction, and PCR amplification are all possible sites of errors. Primer binding, especially in emerging or highly mutagenic strains of a pathogen, or in the case of multiplex PCR, poses certain challenges due to potential cross reactivity with other pathogens. In addition, PCR requires expensive laboratory equipment with high turnaround time in a high volume situation such as in the current pandemic. Ideal sampling techniques are difficult to ascertain as viral loads can vary depending on the stage of infection and sampling location [22]. Wu et al. from the Chinese CDC has reported that upper respiratory samples used for RT-PCR can often be insufficient and provide false-negative diagnoses [23], highlighting the need for alternative detection methods that can be used independently or in conjunction with standard RT-PCR testing. 

The pandemic has evolved since its inception, and with new emerging strains, sequencing for SARS-CoV-2 has been identified as a critical next step to monitor and contain the spread of the virus. The US is lagging in SARS-CoV-2 sequencing efforts and there has been a call from federal public health offices to ramp up sequencing efforts.

## 3. Next-Generation Sequencing (NGS)

NGS is an emergent technology that has the power to sequence billions of nucleic acid fragments simultaneously, with recent advances rendering dramatically reduced time and cost of sequencing. The power of high throughput NGS technology presents the scientific community with many promising applications, and the current pandemic is providing an enormous sense of urgency to push some of these applications into widespread usage. One application opportunity for NGS is in the field of clinical diagnosis of infectious disease. Using NGS technology for the diagnosis of infectious diseases offers an unbiased approach detecting pathogens that does not rely on culturing or the need for clinical hypotheses. While standard testing procedures require clinicians to identify possible explanations for a patient’s symptoms and employ tests aimed at those specific pathogens, NGS testing can reveal the presence of all types of microorganisms present in a sample, including bacteria, viruses, fungi, and parasites. 

The massive additional benefit of the widespread adoption of NGS for routine diagnostics is the wealth of information provided by the test results. Sequencing data are vital information for those involved in the fight against infectious disease, aiding in vaccine and antiviral development, phylogenetic analysis, viral spread tracing, the monitoring of the evolution of a pathogen, the development of other diagnostic tests, and the identification of any primary and intermediate zoonotic hosts [10]. Before examining the implementation of NGS for clinical diagnosis and the SARS-CoV-2 pandemic in the literature, we describe the basic nature of the technology, its bioinformatic processing pipelines, and challenges facing its widespread application.

### 3.1. NGS Fundamentals

NGS technology was developed around the turn of the century as a more advanced and powerful method of sequencing genetic material. Sanger sequencing, first developed in the late 1970s, is the original sequencing method and allowed for the completion of the Human Genome Project in the early 2000s. This early technology is underpinned by a chain-termination process that relies on the addition of fluorescently tagged dideoxynucleotides (ddNTPs) to PCR amplification reactions of the genetic material that is to be sequenced. The ddNTPs lack the 3′-OH group required to form a subsequent phosphodiester bond, and thus function to terminate the growing nucleic acid chain at random points in the elongation stage of synthesis. 

These random termination events result in the accumulation of nucleic acid fragments of varying lengths that can be separated by size via gel electrophoresis. The order of the nucleic acid bases in the DNA or RNA in question is subsequently determined by analyzing the gel. The distinct fluorescent pattern of each different ddNTP (ddATP, ddTTP, ddGTP, and ddCTP) can be measured to reveal which base is present at each band location. The smallest nucleic acid band on the gel travels the farthest and represents the 5′ end of the sequenced strand; from this starting point, the strand is read by moving up the gel towards the 3′ end. The Sanger method of sequencing is still routinely used in common laboratory applications and for the verification of NGS data. 

#### 3.1.1. NGS Methods

The development of NGS subsequently occurred in the early 2000s and rapidly advanced the scientific community’s ability to investigate the genomes of living organisms via its massively high throughput capability. The methods of next-generation sequencing vary in their technical mechanisms, but all share the basic defining features. They rely on samples being broken down into fragment libraries that are each amplified and sequenced independently, generating millions of fragment reads (small sequences) that can be pieced together to generate a readout of the genome. Pyro-sequencing, sequencing by synthesis, sequencing by ligation, and ion-semiconductor sequencing are all subtypes of NGS technology, each coming with its technical variations as well as advantages and weaknesses. Sequencing by synthesis (SBS) is a widely used method and is utilized by several commercial companies. 

In the SBS method, the generated library fragments are modified with adapter motifs which include sequence binding sites, indices, and regions complementary to oligomer linkers on a chip surface. The fragments are subsequently attached to a chip via binding with oligomer linker sequences, and each fragment is then amplified at a distinct point on the chip, creating its cluster. The amplification is accomplished by a process involving bridge amplification, resulting in distinct clusters of fragments with identical sequences, present in both the forward and reverse orientations. The clusters are modified by washing processes so that only the forward sequences remain, and sequencing is commenced. After reading the forward sequences, the process is inverted and the reverse sequences are read. The sequencing process itself is accomplished by successive rounds of the addition of fluorescently tagged nucleotides whose base can be identified in real-time by their light emission profile. The device can analyze each cluster’s data simultaneously, effectively reading millions of fragment sequences in a short amount of time [24].

Different companies use different iterations of NGS technology, an example being the platform developed by Ion Torrent Systems, which uses ion-semiconductor technology. This machine detects hydrogen ions released during the polymerization of a template strand; therefore, it also falls under the header of sequence by synthesis [25]. Detailed descriptions of all NGS systematic processes fall outside the scope of this review.

#### 3.1.2. Bioinformatic Processing of NGS Data

NGS technology generates a massive amount of raw data that requires substantial amounts of computational processing to yield significant and actionable results. An NGS bioinformatic pipeline refers to a series of algorithms that the data are run through to generate a useful interpretable output [26]. Due to the differences among proprietary NGS technological methods and differences in commercial laboratory processes and goals, there is inevitable variation in the software and computational tools utilized by these groups. All NGS bioinformatics pipelines share several major features including sequence generation, assembly and alignment, variant identification, variant annotation, and variant prioritization and visualization [25,26,27]. Bioinformatic pipelines also require rigorous quality control methodology to guarantee the validity of results.

Sequence generation is the process by which the NGS platform converts sensor information into base calls, thereby generating the raw nucleotide sequence of the fragment at each cluster. Technical methods vary among manufacturers. The Illumina sequence-by-synthesis platform accomplishes this task by measuring fluorescence output at each oligonucleotide cluster to identify the nucleotide being incorporated into the strand during each round [25].

The next component of the bioinformatic pipeline involves the assembly and alignment of sequencing fragments. Assembly refers to the creation of *contigs*, which are longer consensus sequences pieced together by computational analysis of overlapping sequences generated from different clusters. Alignment is also described as *mapping* and refers to the arrangement of fragment reads or contigs along a reference genome. The relative ordering of alignment and assembly in the pipeline workflow is variable and depends on individual project aims. The two processes can occur concurrently, or a de novo sequence can be constructed, followed by a comparison to a reference genome. The de novo construction of a genome is a method by which scientists can create a reference genome for newly studied organisms [25].

An important consideration in the alignment step is the concept of stringency, which refers to how strictly the read sequences or contigs must match the reference sequence. Low stringency would allow for possible strand misalignment, while high stringency could allow variants to be lost due to their lack of an exact match with the reference sequence. Short-read sequencing (<250 bp) can also face difficulties in alignment due to the presence of large regions of homology in the reference genome that restrict the ability of short fragments to be appropriately mapped [28].

Variant identification, also known as *variant calling*, is the practice of identifying regions where the sample and reference sequences diverge [26]. The most basic example of this would be a single nucleotide variant (SNV), where one nucleotide is mismatched amid otherwise identical sequences. Other variants include small insertions and deletion mutations (INDELs), copy number variations, or larger structural changes such as inversions or translocations. There are a variety of computational tools available for use in the variant calling step of the NGS bioinformatics pipeline.

Annotation is a process whereby identified variants are further characterized in the context of associated metadata. Essentially, programs query assorted databases to link variants to useful information that helps put them into context. Aspects such as the variant’s location in the genome, predicted changes to cDNA or amino acid sequences, or how commonly they show up in common variant databases are probed [26]. These annotations are subsequently used to prioritize the variants. Insignificant findings can be filtered out to help scientists focus on salient findings; this is important to help researchers or clinicians avoid being inundated with an unmanageable flood of information. An example of an insignificant finding is an SNV that has been widely established as benign [26]. Laboratories can utilize hard filters that remove all variants except for those with relevance to their research or clinical interest. 

#### 3.1.3. Quality Control for Bioinformatics Pipelines

Another universal and vital aspect of all NGS bioinformatic pipelines is quality control. Multiple steps are taken throughout the data processing operation to ensure that the results are of high quality and clinically useful. Some common quality control metrics are described below.

A simple yet important measure of validity is the number of reads performed by the sequencer. Coverage depth refers to the average number of reads there are for a reference locus and helps determine the degree of confidence of the validity of a variant discovery. Horizontal coverage refers to the percentage of the reference genome that is aligned to NGS-generated sequences. Higher coverage lowers the likelihood that a false positive or false negative variant will be obtained [29]. 

When multiple samples are run together or *multiplexed*, each sample is identified by a distinct molecular bar code. Demultiplexing involves the separation of samples by barcode after base calls are made, and the success of this process yields a useful quality metric [25]. During sequence generation, the NGS platform assigns a quality score to each “base call” to indicate the statistical likelihood of correct identification of each nucleotide. Similarly, during the alignment step, a mapping-quality score is assigned to each of the fragment reads and is used as an indication of the likelihood of accurate alignment to the reference genome. The alignment phase allows for the calculation of the horizontal coverage and depth of sequencing, useful indicators of statistical significance, as mentioned previously [25,26]. Before variant calls can be made, more computational processing is often necessary to ensure that the base calling and genome-alignment mapping data are of high quality. An example of one of these prevariant identification processes is local sequence realignment around loci where INDEL mutations are expected to have occurred. *Variant filtering* is a post-variant identification step used to screen identified genomic variants for likely false positives using metadata such as base-call quality scores, mapping quality scores, and read depth, among others [25].

### 3.2. NGS and the SARS-CoV-2 Pandemic

#### 3.2.1. NGS for Pathogen Detection: Prepandemic

The development of sequencing technology for diagnostic and pathogen surveillance was an urgent undertaking even before the SARS-CoV-2 pandemic erupted, as the cause of many infections often goes undiagnosed. Jain et al. found that of 2,488 hospitalized patients with community-acquired pneumonia, only 853 cases (38%) were diagnosed with a causative pathogen [30]. The problem extends beyond the respiratory system; clinicians are often similarly unable to pinpoint the etiology for CNS infections. A study conducted by Glaser et al. of 1570 patients in California found no etiology in 63% of cases of encephalitis [31]. These unexplained infections often lead to inadequate treatment and poor outcomes, while simultaneously contributing to the widespread overuse of antibiotics as unsure providers use antibiotics liberally when an infection is unexplained. 

Using NGS technology for the identification of infectious diseases promises an unbiased approach that does not rely on culturing, and NGS has already been shown in various case reports and preliminary studies to be capable of identifying pathogens in samples taken from the respiratory system, central nervous system, gastrointestinal system, and the eyes [32]. Studies have demonstrated the utility and practicality of NGS in diagnosis, showing that results can be obtained from “sample-to-answer” in 48 h [33], similar to the wait times experienced by those being tested by standard RT-PCR COVID-19 tests around the country. The ability to run many samples together by multiplexing should allow laboratories to accommodate the high number of samples for testing to clear the backlog. Although there is currently limited data available on the use of NGS for high volume COVID-19 testing, we have examined reports from labs across the world that are using NGS technology to aid in the fight against the SARS-CoV-2 virus.
Key Findings:1. A large percentage of commonly clinical diseases are due to infections of unknown etiology [30,31].2. NGS has been proven to be capable of identifying infectious microorganisms from various patient sample types [32].3. NGS has been shown to provide clinically practical turnaround times [33].

#### 3.2.2. NGS for Detection of SARS-CoV-2

In late January 2020, Lu et al. reported SARS-CoV-2 genomic data from nine patients presenting with pneumonia of unknown origin at three hospitals in Wuhan, China [1]. BAL and cultured isolates were used as samples. The patients’ samples were negative for known respiratory pathogens, with five tested by the Chinese CDC and four by the BGI group in Beijing, China. NGS technology was used to sequence and identify the causative pathogen, with the BGI and CDC labs differing slightly in their sequencing techniques and bioinformatic processing pipelines. In both groups, gaps between contigs were connected using Sanger sequencing and terminal genome regions were identified via rapid amplification of cDNA ends (RACE). 

At the BGI group, RNA extraction of BALF samples was carried out with a QIAamp Viral RNA Mini Kit, and a probe-captured technique was used to remove human nucleic acid material. Next, RNA was reverse transcribed to cDNA, second-strand synthesis was performed, and a DNA library was constructed. The DNA library was quantified with a Qubit method and transformed into a single strand circular library. Rolling circle amplification was used to construct DNA nanoballs, and they were subsequently qualified. The DNBSEQ-T7 high throughput sequencer from MGI was used with paired-end, 100 bp read lengths. High quality reads were filtered for human reads against the hg19 human reference genome with Burrow-Wheeler alignment software. The remaining data were aligned with published data on coronaviruses from the US National Center for Biotechnology Information. Mapped reads were assembled with SPAdes software to create a consensus genome sequence.

The Chinese CDC sequencing protocol similarly used the QIAamp Viral RNA Mini Kit to extract viral RNA from the clinical samples, followed by cDNA synthesis and second-strand synthesis. cDNA libraries were generated and then purified with Agencourt AMPure XP beads to remove contaminants. Following quantitation, the sequencing was carried out on MiSeq or iSeq platforms from Illumina. The terminal genome regions were identified by the use of Rapid amplification of cDNA ends (RACE) system from Invitrogen. Assembled genomes were confirmed with traditional Sanger sequencing. The raw sequencing reads were filtered via the same protocol used by the BGI group, and CLCBio software version 11.0.1 was used for de novo assembly, variant calling, and alignment. The bat-SL-CoVZC45 virus (containing 87.99% sequence similarity) was also used to perform a mapped assembly. 

Sequencing yielded eight full genomes and two partial genomes (one patient’s BALF sample was used to isolate the virus, which was also sequenced, yielding 10 total samples). The sequences were used to generate PCR-based assays, that were then used to confirm the presence of the SARS-CoV-2 virus, and cycle threshold (Ct) values ranged from 22.85 to 34.23.

The results of sequencing the viral genome in this study yielded highly useful information during the early stages of the SARS-CoV-2 outbreak. Genomic analyses led to the revelation that, while the whole genome sequence of SARS-CoV-2 is highly similar to bat-SL-CoVZC45 (87.99% similarity) and bat-SL-CoVZXC21 (87.23%), the receptor binding domain (S1) sequence of the spike protein (S), was more similar to that of SARS-CoV, the virus responsible for the first SARS outbreak in the early 2000s. This evidence supports the suggestion that SARS-CoV-2 uses the ACE-2 receptor to gain entry into cells, the same route utilized by SARS-CoV. The utilization of ACE-2 receptors by SARS-CoV-2 has also been demonstrated in infectivity studies by Zhou et al. [3]. The phylogenetic analysis, made possible by the assembled sequences, allowed the classification of the virus, showing that the virus belongs to the subgenus Sarbecovirus, a member of the Betacoronavirus genus. The high sequence similarity (over 99.9%) among viral samples obtained from the nine patients in Wuhan provides evidence of very recent entry into the human population. 

Other laboratories in China conducted parallel investigations at the onset of the outbreak, such as Zhu et al. [4], who used a similar combination of Illumina and nanopore sequencing, RACE, and Sanger sequencing to identify and characterize the SARS-CoV-2 genomes extracted from three patient samples in Wuhan, China. Their bioinformatics pipeline included CLC Genomics software, version 4.6.1; Muscle; and RAxML (13) for phylogenetic analysis. Their sequencing protocol yielded more than 20,000 viral reads per sample, obtaining one full-length genome and two nearly full-length genomes. They similarly noted that contigs aligned with high similarity with bat-SL-CoVZC45. Published 24 January 2020, they reached similar conclusions to Lu et al. regarding the phylogenetic characterizations of the virus and used their de novo generated sequences to design primers for PCR-based diagnostic assays. 

Groups all over the world are now investigating possible diagnostic interventions made possible by NGS technology. Campos et al. reported the use of metatranscriptomic next-generation sequencing technology in the detection of SARS-CoV-2 in a nasopharyngeal swab specimen from a patient in Feira de Santana-Bahia, Brazil [34]. They used the Ion S5 platform from ThermoFisher with an Ion 540™ chip and the Ion Total RNA-Seq kit v2. This platform uses an ion-semiconductor sequencing process, and they implemented the Low Input RiboMinus™ Eukaryote System v2 from ThermoFisher to remove rRNA from one sample. The rRNA-depleted library contained human transcripts as 77.29% of total reads, while the whole RNA library had 84.49% of total reads as human transcripts. Contigs from the rRNA-depleted library provided 29.9% genome coverage, while contigs from the non-depleted sample yielded only 5.4% genome coverage. Total genome coverage from all viral reads in the rRNA depleted sample was 59.9%. These results indicate that rRNA-depletion strategies may play a role in improving NGS diagnostic abilities.

Moore et al. have reported on the use of amplicon- and metagenomic-MiniION-based sequencing in the identification of SARS-CoV-2 and co-infections, respectively [35]. Amplicon-based NGS is a tool that is commonly used to provide highly specific data on the presence of organisms in a sample via primers targeting highly conserved areas of a genome. This is contrasted with metagenomic-NGS which takes a “shotgun” approach, identifying all genetic material in a sample, not just those that contain the highly conserved genetic region. Primers in this amplicon-based approach were designed with sufficient overlap that the sequence of SARS-CoV-2 could be reconstructed from the individual fragments. The study was limited as it only included two patients, both from the UK. Primers were designed for amplicon-based NGS sequencing of SARS-CoV-2 to generate approximately 1000 base pair fragments with roughly 200 base pair overlaps for sequence assembly, and the assay successfully sequenced the SARS-CoV-2 genome in both patients. For validation, they spiked samples with VP35 RNA from Ebola Virus as an internal control. The mapping software successfully identified the internal control RNA and also identified the presence of Fusobacterium periodonticum and human cytomegalovirus (human betaherpes virus 5) in addition to SARS-CoV-2 in the mNGS results. The group used Oxford Nanopore Technology’s (ONT’s) cloud-based pipeline EPI2ME (WIMP rev. 3.2.2) workflow for bioinformatic analysis. Patient 1 was sampled twice (two days apart) and patient 2 was sampled once, yielding total reads of 8,698,559 (78.6% of which were human reads), 9,890,327 (97.7%), and 5,849,966 (92.7%), respectively, during mNGS. The metagenomic approach did not provide uniform genome coverage among the three genomes, and the amplicon-based sequencing method provided a much higher read depth than the metagenomic approach. This study highlights the useful abilities enabled by the hypothesis-free approach taken by NGS. Identifying co-infections via the use of NGS, whether viral or bacterial, is highly relevant to clinical decision making and could help guide treatment and patient outcomes. 

A table comparing currently employed NGS methods—short read: Illumina, Ion torrent, long read: Nanopore WGS assay—for detection of COVID-19 is presented (Table 1).

#### 3.2.3. Co-Infection in COVID-19 Patients

Co-infection data relating to SARS-CoV-2 is of high interest to clinicians around the world, as rates of co-infection differ among viruses, and their presence is associated with poor patient outcomes in many common viruses [36]. One early investigation of 191 inpatients with COVID-19 in China in December 2019–January 2020 reported that 50% (27/54) of non-survivors had a secondary infection [37]. The possibility of co-infection is also important for clinical diagnosis. If co-infection is found to be rare, a positive test for a common respiratory pathogen could indicate a lack of SARS-CoV-2 infection, while higher chances of co-infection would not allow clinicians to rule out such a possibility. Reports on the rates of co-infection in COVID-19 patients have varied widely, ranging from approximately 1–20%, with one early study in Wuhan reporting a co-infection rate of 57.3% [38,39]. Consequently, larger-scale studies are needed to fully assess the role of secondary infections in these patients.

Lansbury et al. published a systematic review and meta-analysis of 30 studies on 3834 COVID-19 patients to assess co-infection data. The studies were conducted mainly in China (23/30), with some from the United States, Spain, Thailand, and Singapore. The studies were also quite heterogeneous in their structures and types of reported statistics, leaving the authors to parse through diverse datasets and draw conclusions cautiously. They found that 7% of hospitalized COVID-19 patients had a bacterial co-infection, while patients in the ICU had bacterial co-infection rates of 14%. The most common bacteria in these patients were Mycoplasma pneumonia (42% of patients with a specific type of bacterial co-infection reported), Pseudomonas aeruginosa (12%), and Haemophilus influenzae (12%). These rates are lower than those seen during more serious influenza pandemics, such as the 2009 H1N1 pandemic, and the authors thus discourage the broad application of antibiotics in COVID-19 patients when not expressly warranted. The authors estimated that 3% of hospitalized COVID-19 patients were infected with an additional respiratory virus, with respiratory syncytial virus (RSV) and influenza A being the most commonly noted co-infections (16.9% and 15.5%, respectively, of patients with the specific type of viral co-infection reported) [40].
Key Findings:1. NGS is useful for yielding essential information about a pathogen at the outset of an infectious outbreak [1,4].2. NGS is capable of being implemented as a diagnostic assay for SARS-CoV-2 infection [34,35].3. NGS is capable of accurately identifying co-infection in COVID-19 patients [35].

#### 3.2.4. NGS as a Tool for Understanding SARS-CoV-2: Additional Benefits of Adoption

The adoption of NGS for widespread use yields the additional benefit of generating massive amounts of data that can be used to study pathogens. Researchers in academia, government, and industry have also been using sequencing technology and data to aid in understanding the attributes, processes, and phylogenetics of the SARS-CoV-2 virus. Discoveries in these areas are important for the development of vaccines, antivirals, and novel diagnostics, as well as generating useful information for public health authorities such as data on transmission and viral tracing. The widespread adoption of NGS in diagnosis will lead to major gains in scientists’ and governments’ ability to monitor emerging variants of infectious diseases such as SARS-CoV-2. The United States CDC is leading what it calls the “SARS-CoV-2 Sequencing for Public Health Emergency Response, Epidemiology, and Surveillance (SPHERES)” program. The program is aimed at coordinating large-scale sequencing of the SARS-CoV-2 virus to facilitate these previously mentioned goals [41].

#### 3.2.5. Understanding Physical and Chemical Properties of the Virus

As previously noted, Lu et al. used sequencing results generated in their lab to provide evidence that the structure of the receptor binding domain (S1) of the spike (S) protein was highly similar to that of the original SARS-CoV virus [1], indicating that the protein would likely bind the ACE-2 receptor. This type of genetic analysis is a highly useful byproduct enabled by sequencing infectious diseases, as it can reveal structural parameters that guide pharmaceutical developments. 

Kim et al. used NGS technology to provide a high resolution readout of the SARS-CoV-2 transcriptome and epi-transcriptome using viral RNA isolated from a patient in South Korea, revealing a complicated array of RNA transcripts and RNA modification sites [42]. They used a combination of sequence-by-synthesis (SBS) and Nanopore-based direct RNA sequencing (DRS) methods to map the full-length SARS-CoV-2 genome (gRNA) as well as sub-genomic RNAs (sgRNAs) which code for structural proteins, open reading frames (ORFs), and transcription regulatory sequences (TRSs) of the SARS-CoV-2 transcriptome. DRS techniques enable long reads of RNA without conversion to cDNA, allowing RNA modifications to be observed. The group generated in vitro RNAs as negative controls to study the modifications made to the virus inside of cells. Differences in ion current were noted at 41 sites between the patient-isolated and negative-control viral genomes, pointing to likely sites of RNA modification, and cross-examination revealed that “AAGAA” motifs were commonly associated with these modification sites. The group developed in-house software to analyze DRS sequencing data, and this software was used to measure the poly-A tails of full-length gRNAs and sgRNAs. They found that gRNAs have longer poly-A tails, than sgRNAs, and that sgRNA poly-A tails have two distinct populations, one with ~30 nts and one with ~45 nts. These differences likely result from age of the RNA and could indicate strategies for viral RNA degradation. They also noted that modified RNA molecules had shorter Poly-A tails. The authors speculate that these modifications could play roles in viral RNA stability control or evasion of host immune response. This study provides rich information on the complex intracellular processes involved in SARS-CoV-2 infection and could lead to breakthroughs in antiviral development.

Coutard et al. analyzed SARS-CoV-2 sequencing data and identified a furin-like cleavage site present in the S protein amino acid sequence that is not present in coronaviruses of the same clade [43]. They proposed that the addition of this cleavage site in the amino acid structure of the S protein may have been a gain of function mutation that allowed for efficient spread into the human population. They supplement their hypothesis with evidence that furin expression levels are high in the lungs and that host cells attempt to inhibit the activity and availability of furin-like enzymes during viral infections [44]. The authors note that the presence of similar furin and furin-like cleavage sites have been linked to higher pathogenicity in infectious bronchitis virus, influenza viruses, and other human coronaviruses. 

Anand et al. provided supporting evidence on the importance of this furin-cleavage site [45]. They performed computational analyses on 10,967 SARS-CoV-2 genomes available in the GISAID public database, and report that the furin cleavage site on the spike (S) protein is identical to the furin cleavage site present on the human epithelial sodium channel alpha subunit (ENaC-a). The EnaC-a subunit requires cleavage by furin proteases for activation in the same manner as the S protein of SARS-CoV-2, and the EnaC channel is present in high levels in regions of initial SARS-CoV-2 infection (respiratory epithelium, nasal cavity, etc.). They hypothesize that the furin cleavage site present on the SARS-CoV-2 virus could compete for activation with the EnaC-a furin cleavage site, causing dysregulation of cellular electrolyte balance, leading to the high levels of fluid found in the lungs of some COVID-19 patients. Taken altogether, this analysis of sequencing data provides another interesting proposal that could point to an antiviral candidate for SARS-CoV-2 and shed light on the high transmission capability of the novel virus.

In Japan, Wakida et al. have developed a technique that they call “Fate-Seq” which uses next-generation sequencing to identify viral RNA sequences that can help ensure RNA stability inside host cells [46]. The study found that the original SARS-CoV virus, first seen in the early 2000s, contains 21 RNA sequences that could confer stability to viral RNA inside host cells, and comparative analysis with the SARS-CoV-2 genome shows high levels of conservation in these sequences. These findings may point to mechanisms that the novel SARS-CoV-2 virus uses to inhibit host RNA degradation systems and promote viral replication. 

Yadav et al., researchers in India, used the Illumina MiniSeq platform to sequence three initial positive SARS-CoV-2 samples discovered by RT-PCR testing in February of 2020 [47]. One of the sequences was of low data quality and so was excluded from the analysis. Extraction was performed with the QIAamp Viral RNA kit followed with Qubit RNA High-Sensitivity kit for quantification. Libraries were prepared and quantified with KAPA Library Quantification Kit, and CLC genomics workbench version 11.0 was used for bioinformatics analysis. For case 1, 20,096 viral reads were obtained from 5,615,846 total reads for a reconstructed SARS-CoV-2 genome of 29,854 nucleotides (99.83% coverage). Case 3 provided 11,296 viral reads out of 1,405,038 total reads for a reconstructed genome of 29,851 nucleotides (99.83% coverage). 

They used these data to identify predicted linear and conformational B-cell epitopes as well as T-cell epitopes using a multitude of software products that predict amino acid structures based on sequencing data. These epitopes represent ripe targets for vaccine development and warrant further investigation. The sequence data from the two complete genomes showed 0.04% nucleotide divergence and 0.10% amino acid divergence, and phylogenetic analysis was able to indicate that these patients represented separate introductions into the country. Additionally, of note is that blood samples from these three confirmed COVID-19 patients were negative, highlighting the need for the development of accurate testing assays. This work reiterates the value of sequencing data in helping public health authorities gain information that can help them make management decisions.
Key findings made possible by NGS technology1. The presence, structure, and function of the SARS-CoV-2 spike protein [1,43].2. The presence and ramifications of a furin-like cleavage site on the SARS-CoV-2 spike protein [43,45].3. Mechanisms of viral stability inside human cells [42,46].4. The presence and structure of B-cell and T-cell epitopes on viral proteins [47].

#### 3.2.6. SARS-CoV-2 Phylogenetics and Mutational Characteristics

Scientists in Italy, a country especially hard hit by the outbreak, began performing NGS analyses early in the pandemic to gain a better understanding of how the virus has spread across the country. Lorusso et al. sequenced 46 samples from patients in the Abruzzo region of central Italy between 16 March and 23 March 2020 [48]. They chose these 46 samples for NGS among 839 SARS-CoV-2 positive samples based on their low Ct scores during RT-PCR testing. Their protocol utilized the MiniSeq Mid Output Kit (300-cycles) with 150 bp paired-end reads, and they used trimmomatic bowtie2 and samtools software products for bioinformatic processing.

Forty-five out of 46 NGS-generated sequences were of high read quality and suitable for analysis, while 16/45 had horizontal coverage >95.2% and were deposited in the GISAID database. Coverage depth for these 16 sequences ranged from 87× to 3721×. All sequences generated were >99% similar to the Wuhan-Hu-1 reference strain, yet all contained single nucleotide polymorphism (SNP) mutations. Phylogenetic analysis of these genomes tentatively points to two separate modes of introduction to the region. Twenty-nine out of the 45 sequences showed R203K and G204R mutations in the N protein, while 13 did not (3 were partial genomes and were missing this portion of the genome). These N protein mutations have been associated with Northern Europe, but a lack of sequences from the outbreak in northern Italy makes it difficult to draw robust conclusions at this time. More studies monitoring mutational data will help researchers trace routes of viral spread, allowing authorities to better stop outbreaks in the future, and they will also be vital to ensure that vaccine, antiviral, diagnostic primer–probe designs are up to date and adequate for an ever-changing virus. Monitoring viral mutation also helps researchers understand mechanisms of the virus, such as those of transmissibility and pathogenicity.

Several groups have reported on the mutational characteristics of the SARS-CoV-2 genome, including van Dorp et al. who have assessed 7666 public genome assemblies [49], commenting on the relatively non-conservative nature of the N and S genes in the viral genome. The group has identified 198 recurrent mutations or mutations that have emerged independently multiple times, with 80% of these being non-synonymous mutations. More than 15 recurrent mutations were noted in each of the Nsp6, Nsp11, Nsp13, and S protein regions, respectively. Thus, these regions of the viral genome are non-conservative, as shown in similar studies.

In one such study, Wang et al. submitted a comprehensive analysis of 6156 SARS-CoV-2 genomes obtained between 5 January and 24 April, providing a breakdown of mutation patterns in the viral genome as well as across geography and time [50]. The group assessed the genomes for analysis of their geographical distribution by k-means clustering. They noted that genomes in the study, gathered from across the world, cluster into five main groups with common mutations as shown in Table 2. Note that these are not the only mutations present in the genome of each virus, just those that members of a cluster have in common; there can be subtypes of these groups themselves. From these clusters, some information can be drawn. Clusters 1 and 2 were apparent in the early data and are the dominant subtypes found in Asian countries, which is intuitive as the virus originated in Asia and subsequently spread across the world, accumulating more mutations along the way. The authors also note that the 23403A > G mutation (D614G amino acid change) found in clusters 3–5 is a spike protein mutation and could be a contributing factor to the high levels of spread seen in Europe and the United States. All of the five groups can be found at some level in the United States, and the authors went further to classify the SARS-CoV-2 genomes found in the US into three major clusters (A, B, and C). Cluster A is spread out across the nation, although in somewhat smaller numbers. Cluster B is highly prevalent on the US west coast, especially in the state of Washington, while the east coast shows a high prevalence of Cluster C. This distribution provides some evidence that the east coast COVID-19 outbreak originated mainly from Europe, as Cluster C is a descendant of Cluster 3 from the world-wide data. 

The mutations were also assessed for their protein alterations and a mutation ratio and mutation h-index were determined for each genomic region. The mutation ratio reflects the absolute number of mutations found in the data relative to the length of the region (i.e., number of mutations found divided by the number of codons, or residues, in that region). This number reflects the relative conservative or non-conservative nature of each region. The mutation h-index is also provided to account for the fact that some mutations occur many times while others appear only a handful of times in the data; this index is mathematically defined as “the maximum value of *h* such that the given protein genetic section has *h*single mutations that have each occurred at least *h*times” [50]. Their calculated mutation ratios and h-indices for a given genomic region were highly correlated with each other. When assessing these values together, it is noted that the most conservative regions of the viral genome are, in order, the envelope € protein, main protease, and endoribonuclease. Alternatively, the least conserved regions were the nucleocapsid (N) protein, Spike (S) protein, and papain-like protease. 

Several prominent mutations appeared early in the pandemic with high frequency, including D614G (nt 23,403) in the S-protein and P323L (nt 14,408) in the RNA-dependent RNA polymerase (RdRp) protein, among others. As noted, the authors speculate that the D614G mutation, present in clusters 3–5, may confer a higher transmission ability to the virus, and also mention that it and other’s proximity to epitope regions may be relevant to vaccine development. Pachetti et al. analyzed 220 SARS-CoV-2 genomes obtained from patients across the world from December 2019 through mid-March 2020 and investigated mutations in the RdRp gene, including the P323L (nt 14,408) mutation [51]. RdRp is involved in viral replication, and thus likely plays a role in the generation of new mutations in the viral genome. A silent mutation in this region appeared in their data on 9 February in the UK (nucleotide position 14,408), while the non-synonymous P323L mutation appeared on 20 February in Lombardy, Italy. They divided the genomes obtained after 9 February into groups that either had the 14,408 mutations (*n* = 53) or did not (*n* = 84) and found that those with the RdRp mutation had a statistically significantly higher number of mutations, and a median number of point mutations of three versus one, respectively (*p* < 0.001). These data suggests that an RdRp mutation could confer higher mutation rates by interfering with viral proofreading abilities or by some other mechanism. 

Chen et al. have reported on the effect of S-protein receptor binding domain (RBD) mutations on viral infectivity [52]. They used a computational approach to estimate the changes in binding affinity between the viral S-protein RBD and the human ACE-2 receptor that occur following mutations found in 13,752 SARS-CoV-2 genomes available in the GISAID database. They assessed the five clusters of genomes presented by Wang et al., with the addition of a small sixth cluster, and found 55 amino acid mutations on the RBD. After evaluating the presence and frequency of each RBD mutation in each cluster, they determined that the mutational patterns of the RBD in five out of the six genome clusters had evolved towards higher RBD-ACE2 binding affinity (except for cluster 3). The authors implied that this points towards a trend of increased infectivity in the SARS-CoV-2 virus, but these results are limited in that only the receptor binding domain of the Spike protein was assessed. Other areas of the S-protein or other unknown factors could play important roles in viral infection.

Shen et al. used metatranscriptome NGS technology to evaluate mutational properties of the SARS-CoV-2 virus in eight BALF samples from patients in Wuhan [53]. Using an Illumina HiSeq 2500/4000 platform, they searched for intra-host variants (varying strands of the virus present within individual humans) using a multitude of bioinformatic software applications. Variants had to meet rigorous inclusion criteria to be fit for analysis, including: “(1) sequencing depth ≥ 50, (2) minor allele frequency (MAF) ≥ 5%, (3) MAF ≥ 2% on each strand, (4) minor allele count ≥ 5 on each strand, (5) minor allele supported by the inner part of the read (excluding 10 base pairs on each end), and (6) both alleles identified in ≥3 reads that specifically mapped to the genome of Betacoronavirus [53]”. Sequence depth ranged from 18× to 32,291×, and five samples had 50× depth or greater on >80% of their genome.

The study reported a median number of intra-host variants to be four with a range of 0–51, but the transmission of these intra-host variants was not observed, indicating that a bottleneck may be associated with the transmission, although more publicly shared sequences are needed to make broad generalizations. The authors note that high numbers of variants present in individual patients may increase viral fitness, making eradication more difficult. Chronically infected individuals could provide opportunities for the virus to improve its evolutionary fitness, but the biological significance of high mutation rates in some individuals is still unknown and a source for further study.

In addition to using sequencing technology to study the SARS-CoV-2 virus, genomic sequencing technology can also be applied to the patients themselves to draw conclusions about the virus. Ellinghaus et al. recently released a genome-wide association study identifying two genetic regions in humans that appear to be associated with severe complications from SARS-CoV-2 [54]. Variants at the ABO blood locus and a region of chromosome 3 both were found in higher proportions in patients with respiratory failure, which they defined as requiring mechanical ventilation or supplemental oxygen. The study assessed 1610 COVID-19 patients with respiratory failure from Spain and Italy and 2205 healthy controls. Patients with A-positive blood types were 1.45 times more likely to have severe COVID-19 complications, while those with type-O blood enjoyed a protective effect, being only around two-thirds (0.65) as likely to have similar problems. The region of chromosome 3 contains several relevant genes. SLC6A20 codes for an amino acid transport protein that interacts with ACE-2, while two other genes in the region code for CCR9 and CXCR6 cytokine receptors, respectively. These genes are sites for urgent investigation as the cytokine receptors are involved in the human immune system, and the SLC6A20 gene’s interaction with ACE-2 indicates a high likelihood that it is involved in viral transmission.
Key findings made possible by NGS technology1. Information on the spread of SARS-CoV-2 into and across national borders [47,48,50] and identification of the emergence of distinct viral clades throughout the world [50].2. Mutational rates and characteristics of distinct regions of the SARS-CoV-2 genome [49,50,52].3. Information on important individual mutations that have an outsized impact on the continued spread of the virus including the D614G and P323L mutations [50,51].4. Analysis of intra-host SARS-CoV-2 variants [53].5. Implications of specific human genotypes on susceptibility to developing severe symptoms made possible by human genome sequencing [54].

With the recent discovery of several new variants throughout the world, the need for more ubiquitous sequencing is ever more urgent. In India, there has been a recent surge in new cases along with the identification of a new variant, B.1.6.1.7. Previously, India’s cases peaked in September of 2020 with around 90,000 new cases per day, which began to decline in the following months to numbers nearing 10,000 cases per day. However, with the identification of the new variants, from March 2021 onward there has been a drastic increase in new cases that now exceed 250,000 cases per day. Further, a variant discovered to be spreading rapidly in UK, lineage B.1.1.7 [501Y.V1], has been the source of much media coverage. Models have suggested that this lineage spreads 56% more rapidly than other SARS-CoV-2 lineages [55]. This new strain represents an increasing share of global infections at a point when the virus is already widespread, which suggests that its fitness and/or transmissibility has outmatched that of its peers [56]. On 18 December 2020, the South African government announced a similar emergent strain of the virus, called is 501Y.V2.

The new strains of SARS-CoV-2 share an N501Y amino acid mutation on the RBD region of the spike protein, in addition to many other changes present in these viral lineages. The 501Y.V1 variant contains eight changes to the spike protein alone, while the 501Y.V2 variant has nine such changes. One mutation found in the spike protein of the 501Y.V2 variant, E484K, was identified in a preprint on 28 December 2020, as a possible mutation that could confer an immunological escape to SARS-CoV-2 [57]. 

Thus, screening for mutations and identifying any new strains is critical in the ongoing battle to contain the pandemic. Unfortunately, there has been a lag in screening the phylogentic evolution of the SARS-CoV-2 genome, including in the US, primarily because the resources were aimed at qPCR-based COVID-19 diagnostics. The identification of new strains and upsurge of cases in several parts of the world has led to the adoption of NGS technology on a larger scale. The laboratories testing for SARS-CoV-2 using qPCR have started to either sequence or send out limited number of cases for sequencing to national laboratories for variant monitoring in the US. Though this is an important initial step, there is a need for more laboratories to incorporate NGS technology for variant monitoring, as the process is efficient if all positive cases get screened for variant detection.

Overall, scientists are optimistic that currently available vaccines will be able to suppress the spread of SARS-CoV-2, but these changes are worrisome as the currently available vaccines utilize the spike protein as a way to produce an immunogenic effect in patients. Studies are urgently needed to assess the effect of vaccines against these emerging strains. Thus, there is an urgent need for surveillance testing by NGS to provide a means to keep track of the mutations in the SARS-CoV-2 genome. 

### 3.3. Challenges Related to NGS Adoption

The opportunities made available by the widespread adoption of NGS in clinical laboratories are numerous. However, several challenges impede the routine use of NGS in these settings. To implement NGS in clinical laboratories, validation studies with diagnostic performance characteristics such as limit of detection, sensitivity, and specificity for intended microorganisms need to be determined. Apart from validation, logistical challenges include the management of contamination and the implementation of rigorous, standardized controls at all stages of the testing workflow. Furthermore, challenges exist in ensuring database validity and assessing the clinical significance of results. Finally, cost–benefit analyses must be considered relating to the economic realities of which testing methods are most appropriate in different instances. 

#### 3.3.1. Contamination

Contamination is a persistent concern in clinical microbiology, and the wide-ranging detection ability of the NGS technology can exacerbate this issue. Contamination can be traced to laboratory environments, reagents, or personnel, as well as non-traditional sources of contamination stemming from the nature of NGS technology. These challenges can be addressed with standard sampling and laboratory sanitation protocols, as well as the implementation of standards in the bioinformatic workflow, such as those that require minimum pathogen abundance thresholds to reduce false positives. 

Studies indicate that when an mNGS sample is a true negative, false-positive readings from background contaminants may increase [58]. The NGS limits of detection for causative pathogens can be monitored with positive controls that are spiked into samples, and spiked controls may also help to provide some level of background suppression [59]. Further investigation into the balance between positive controls, background contaminants, and their effects on performance is warranted to eliminate the existing challenges.

The unique nature of next-generation sequencing also yields contamination in downstream processes of the clinical workflow, including data analysis. When samples are multiplexed, some sequences can be misclassified during the de-multiplexing stage to an incorrect index (index hopping), leading to inaccurate test results if not properly addressed during bioinformatic analysis [60]. Additionally, with the possibility of over 99% of reads being from the human host [61], massive amounts of host genetic material, as well as that of the healthy, host microbiome, can complicate the interpretation of results and need to be removed from the dataset to prevent “contamination” of bioinformatic analyses. Finally, errors in reference databases can cause problems for specimen identification, and these databases need to be routinely monitored by regulatory authorities to maintain reliability and up-to-date information.

#### 3.3.2. Expertise

Another issue facing the scalability of next-generation sequencing in routine pathogen detection is the high level of necessary human expertise. The interpretation of results generated by mNGS can be complex and their applicability to clinical decision making is another issue altogether. One way that this issue is being addressed is by the establishment of review committees to interpret results [62]. These committees are analogous to “tumor boards” used in oncological settings and serve as a helpful bridge between complicated data and clinical guidance. Assembling such committees presents a hurdle for many laboratories without the resources of a large institution. 

Review committees are necessary at present due to the lack of established literature on the applicability of mNGS results to clinical care. Examples of questions that face these interpreters include how many reads are needed to provide a true positive result, and if the test’s limit of detection differ among pathogens. Possibly the most difficult questions concern whether a detected pathogen is contributing to the patient’s disease state. During the SARS-CoV-2 pandemic, the requirement of human expertise is greatly exacerbated as the demand for testing outstrips the availability of professionals with the knowledge and skillsets to conduct NGS assays. Over time, accumulated literature, standardized practices, and regulatory guidance should ease these burdens.

#### 3.3.3. Difficulty in Validation

The validation of a pan-pathogenic detecting NGS assay is a difficult logistical undertaking due to the difficulty in obtaining numerous samples of all known pathogenic organisms. Steve Miller and Charles Chiu, in their point-counterpoint review published in the Journal of Clinical Microbiology, advocate for a “representative organism” approach, whereby singular species are used as model organisms to represent the assay’s ability to detect organisms within their respective categories [62]. This approach could help ease the validation burden by providing an initial proof of concept. A reference database could be built over time to include all species detected by the assay with confirmation by traditional, targeted detection methods. An mNGS workflow has already been created and validated for the detection and diagnosis of pathogens causing meningitis and encephalitis from cerebrospinal fluid [63].

Validation studies need to establish basic information such as the necessary ratio of host reads in the sample compared to the negative control, the number of reads needed for a reportable positive result for each pathogen type, and the total number of quality reads needed for acceptable analysis, among other metrics such as limit of detection, sensitivity, and specificity of the assay. Analysis of these validation studies are also difficult because most validation studies use frozen patient samples with known qualities to assess testing performance. Meanwhile, these data are being used as guidance to create mNGS workflows that are to be performed on freshly taken samples, restricting the studies’ ability to appraise specimen stability and variability across time [62]. Regulations are already in place for the use of mNGS technology in oncological settings, thus there is a roadmap in place for scientists to adapt the validation process to infectious disease diagnostics.

#### 3.3.4. Cost–Benefit Analysis

The implementation of mNGS for infectious disease testing comes with significant financial investment at the present moment, including the hiring of expert personnel, procurement of necessary equipment, costs of data storage and security, and the costs associated with performing a test validation study. As previously noted, expert personnel are required for the interpretation of mNGS results in a clinical setting as well as for bioinformatic analysis. The cost of retaining such employees and the upfront investment in testing technology is an overwhelming financial burden for many laboratories. Additionally, the data generated during sequencing require storage capability, and adequate security is necessary due to the HIPPA-protected data therein [62]. Additionally, in the current preregulatory environment, laboratories must perform their validation studies of testing workflows, which incur more expenses. The cost solely for the supplies necessary to complete a validation study of NGS for cerebrospinal fluid would eclipse USD 100,000 [64]. Many of these hurdles will decline in the future as scientific, bioinformatic, and regulatory advances reduce costs associated with per-sample sequencing, the manual burden of interpretation, and the burdens of validation, among others.

Wilson et al. reports that mNGS used for detecting infections in CSF samples could provide a diagnosis for some 3–6% of patients that would not be found by conventional diagnostics [65], but presently the high costs associated with routine mNGS administration in these patients warrant a discussion of the incremental gains provided alongside substantial increases in expense. A solution to this dilemma will likely be that outcome studies are used to determine certain patient populations or conditions where the use of mNGS is more likely to yield an actionable finding [62]. Actionable results can provide substantial cost savings by preventing unnecessary care or avoidance of serious fallout from misdiagnosis. A case report published by Wilson et al. is an illuminating example of how routine NGS testing could help patients, providers, payers, and hospital systems. They describe a 14-year-old boy presenting multiple times with progressive neurological symptoms who eventually entered a medically induced coma after all standard diagnostic work-up was inconclusive, including a brain biopsy. After entering into a coma, informed consent was given by his guardians to be enrolled in an mNGS pathogen detection study that subsequently revealed a Leptospira infection in his CSF, a rare but treatable condition. He was appropriately treated and discharged 32 days later in good condition [33]. Had NGS been a part of his diagnostic work-up earlier in the process, untold amounts of resources, time, and suffering could have been prevented. NGS implementation can thus become financially beneficial in these instances where an early, correct diagnosis is vital, with the savings from a few successful cases making routine use profitable for all involved parties. A comparison of cost, time from sample to reporting, and sensitivity of different common testing modalities is listed in Table 3.

## 4. Conclusions

The review discusses the obstacles and opportunities facing the application of next-generation sequencing technologies for the diagnosis, surveillance, and study of SARS-CoV-2 and other infectious diseases. Technological innovation in the healthcare field faces higher scrutiny than other industries with good reason, but eventually, these barriers can be resolved with proper studies and trials, technology improvements, and regulatory guidelines provided by relevant authorities. The SARS-COV-2 outbreak is providing an impetus for a massive mobilization of research firepower that has led to the rapid adoption of the NGS technology, to meet the urgent challenge of tracking variants in the SARS-CoV-2 genome in an attempt to curtail the spread of the virus and monitor vaccine response.

## Figures and Tables

**Table 1:** Comparison of selected Illumina, Ion torrent, Nanopore WGS assay for detection of COVID-19 as claimed by respective vendors.

Parameters	Illumina COVIDSeq Test	Ion AmpliSeq™ SARS-CoV-2	Oxford Nanopore Technologies
Sample and Systems	1536 to 3072 results can be processed on the NovaSeq 6000 system in 12 h using two SP or S4 reagent kits or 384 results in 12 h using the NextSeq 2000 or the NextSeq 500/550/550Dx (in RUO mode) HO reagent kit	3 samples (Ion 510™ Chip) to 130 samples (Ion 550™ Chip)	12 to 2304 samples using MinION to PromethION
Amplicon Size	400 bp	125–275 bp	_
Limit of Detection	<500 copies/mL	20 copies/reaction	10 copies/reaction
TAT	~24 h	~24 h	~9 h

**Table 2:** SARS-CoV-2 genomic clusters as reported by Wang et al. [50]—worldwide data.

Cluster	Mutations
1	[8782C>T] [28144T>C]
2	[14408C>T]
3	[3037C>T] [14408C>T] [23403A>G]
4	[3037C>T] [14408C>T] [23403A>G] [28881G>A] [28882G>A] [28883G>C]
5	[241C>T] [3037C>T] [14408C>T] [23403A>G] [25563G>T]
US data	
A	[11083C>Y]
B	[17747C>T] [17858A>G] [28144T>C]
C	[241C>T] [3037C>T] [14408C>T] [23403A>G]

**Table 3:** Comparison of common testing modalities.

	Time to Perform Assay	Limit of Detection(Viral Copies/uL)	Infection Status	Coinfection Identification	Ability to Detect Presence of Variant Strains **	Ability to Provide Sequencing Data for Scientific Study
qPCR	4–6 h	0.1–3.16 [13,66]	Active	If organism is actively targeted	Usually	No
NGS	12–18 h	0.125–1 [67,68]	Active	Yes	Yes	Yes
Serology	Variable	Sensitivity: 93.3–100% [69]	Persistent/Resolved *	If organism is actively targeted	Usually	No

* IgM slowly rises during week 1 of infection, peaking at 2 weeks before falling to low levels. IgG levels are usually detected at 1 week, remaining elevated for an extended period [69]. Humoral and Cell-mediated Immunity to SARS-CoV-2 are the subject of much present research. ** qPCR and serology are susceptible to decreasing sensitivity in the face of viral mutations, while mNGS is able to sequence any variant genomes present in a sample. Cost is unaddressed in this table due to the complexity and variability of spending on healthcare in the US. At the moment, qPCR is a less expensive alternative to NGS.
